# An oligo-based microarray offers novel transcriptomic approaches for the analysis of pathogen resistance and fruit quality traits in melon (*Cucumis melo *L.)

**DOI:** 10.1186/1471-2164-10-467

**Published:** 2009-10-12

**Authors:** Albert Mascarell-Creus, Joaquin Cañizares, Josep Vilarrasa-Blasi, Santiago Mora-García, José Blanca, Daniel Gonzalez-Ibeas, Montserrat Saladié, Cristina Roig, Wim Deleu, Belén Picó-Silvent, Nuria López-Bigas, Miguel A Aranda, Jordi Garcia-Mas, Fernando Nuez, Pere Puigdomènech, Ana I Caño-Delgado

**Affiliations:** 1 Molecular Genetics Department, Centre for Research in Agricultural Genomics CRAG (CSIC-IRTA-UAB), Barcelona (08034), Spain; 2 IRTA, Plant Genetics Department Centre for Research in Agricultural Genomics CRAG (CSIC-IRTA-UAB), Cabrils (08348), Spain; 3 Departamento de Biología del Estrés y Patología Vegetal, Centro de Edafología y Biología Aplicada del Segura (CEBAS)-CSIC, Apdo. correos 164, 30100 Espinardo (Murcia), Spain; 4 Instituto de Conservación y Mejora de la Agrodiversidad Valenciana (COMAV-UPV), CPI, Ed.8E, Camino de Vera s/n, 46022 Valencia, Spain; 5 Research Unit on Biomedical Informatics (GRIB), Experimental and Health Science Department (Universitat Pompeu Fabra) Barcelona (08080), Spain

## Abstract

**Background:**

Melon (*Cucumis melo*) is a horticultural specie of significant nutritional value, which belongs to the Cucurbitaceae family, whose economic importance is second only to the Solanaceae. Its small genome of approx. 450 Mb coupled to the high genetic diversity has prompted the development of genetic tools in the last decade. However, the unprecedented existence of a transcriptomic approaches in melon, highlight the importance of designing new tools for high-throughput analysis of gene expression.

**Results:**

We report the construction of an oligo-based microarray using a total of 17,510 unigenes derived from 33,418 high-quality melon ESTs. This chip is particularly enriched with genes that are expressed in fruit and during interaction with pathogens. Hybridizations for three independent experiments allowed the characterization of global gene expression profiles during fruit ripening, as well as in response to viral and fungal infections in plant cotyledons and roots, respectively. Microarray construction, statistical analyses and validation together with functional-enrichment analysis are presented in this study.

**Conclusion:**

The platform validation and enrichment analyses shown in our study indicate that this oligo-based microarray is amenable for future genetic and functional genomic studies of a wide range of experimental conditions in melon.

## Background

Cucurbits, comprising up to 750 different species distributed in 90 genera, are among the most important horticultural crops worldwide [1]. Species of this family have been independently and repeatedly domesticated by different cultures in both the Old and New World, either for food or as materials for a range of products. As a result, they display an enormous, and mostly untapped, genetic diversity.

Melon (*Cucumis melo*) belongs to this family and is one of the most important fleshy fruits for fresh consumption in temperate, subtropical and tropical regions [1]. It has been classified into two subspecies, *C. melo *ssp. *agrestis *and *C. melo *ssp. *melo*, with India and Africa as their centers of origin, respectively [2,3]. Melon is a diploid species, with a basic number of chromosomes x = 12 (2x = 2n = 24) and an estimated genome size of 450 to 500 M [4], similar in size to the rice genome (419 Mb) [5,6] and about four times the size of the Arabidopsis genome (125 Mb) [7].

Based on its relatively small genome, wide morphological, physiological and biochemical diversity and the ability to produce hybrids between unrelated cultivars, *C. melo *has a great potential for becoming a genetic model species [3,8]. Classically, melon and other cucurbits have been used to analyze the development of plant vasculature and its role in the transport of macromolecules [9,10]. Recently, the development of novel genetic and genomics tools, such as a high quality genetic map [11] a draft of a physical map (P. Puigdomènech, unpublished), a TILLING platform (J. Garcia-Mas, unpublished) and an EST collection [1] have enabled the identification and study of genes with agronomic interest in this species. Together, these tools fostered the use of melon as an experimental system to dissect biological processes of economic relevance, such as flavour and textural changes that take place during fruit ripening [12], and the interactions between melon and its pathogens [13,14]. So far, these studies have followed a gene-by-gene approach. As an example, the recent discovery of an ethylene biosynthetic enzyme, CmACS-7, that is required for the development of female flowers in monoecious lines, revealed a novel mechanism involved in organ development [15] and brought up the necessity for developing new tools for high-throughput analysis of gene expression in melon.

Partial sequencing of cDNA inserts of expressed sequence tags (ESTs) have been used as an effective method for gene discovery [1]. In the last decade, the development of several EST collections has opened the way to functional genomic studies in several plant species [16,17]. In addition, EST collections are good sources of simple sequence repeats (SSRs) and single-nucleotide polymorphisms (SNPs) that can be used for creating saturated genetic maps [18,19]. Microarray technology has demonstrated the power of high-throughput approaches to unravel key biological processes [20,21]. cDNA-based microarrays are specially relevant for crop species where little genome information is available both to study a particular biological process or to identify candidate target genes for breeding [22]. Whereas full-length cDNA microarrays were the first choice for the generation of microarray platforms, oligonucleotide-based chips are gradually gaining importance due to the reduction of manipulation steps and their ability to differentiate similar members of gene families [23]. Although cDNA arrays are known to have higher precision across technical replicates, oligonucleotide-based platforms show a greater dynamic range in the evaluation of expression levels [24]. Microarray platforms can be classified with respect to their accuracy and precision [25,26]. The precision of the microarray can be assessed by comparison between different replicas. Accuracy, on the other hand, requires knowledge of the expression of particular genes in the biological system under study [25,26].

In this study, a dataset of 33,418 high-quality melon ESTs obtained from nine MELOGEN normalized cDNA libraries and another available collections from different melon cultivars, corresponding to various tissues in different physiological conditions have been used to generate the first oligonucleotide-based microarray platform in melon. Similar microarray platforms have been generated in other important crop species such as watermelon using 832 EST-unigenes [27], citrus with 21,081 putative unigenes [28], pea from 2,735 ESTs [29] and canola using 10,642 unigenes [30]. However, this high-density microarray is estimated to represent a significant portion of the whole melon transcriptome [1], offering new possibilities for the study of multiple transcripts in a variety of conditions.

In this study, we describe the design and construction of the melon microarray platform, and we validate our tool by means of the analysis of global changes in gene expression profiles in response to pathogen infection and during fruit ripening, taken as model experiments. Our results show that this melon microarray is suitable for the study of global changes in gene expression in different scenarios, until the full genome-sequencing project becomes a reality.

## Results and discussion

### Microarray design and experimental datasets

A dataset of 17,510 unigenes from different melon cDNA libraries was used to generate the probes for the microarray (Table 1). The unigenes used to generate each probe are primarily expressed in fruits (24%), in tissues infected with pathogens (40.6%) and derived from a set of different healthy melon tissues [1]. Probes for the microarray were designed as described in Nimblegen^® ^protocols [31,32] following quality rules such as length (60 mers), non-repetitiveness thus uniqueness, frequency in the transcriptome and melting temperature (Tm). Nimblegen^® ^technology provides long oligonucletides which has been shown to improve sensitivity and discrimination in microarray experiments [31,32]. In turn, fewer probes are required per gene to achieve consistent expression results. The chip design is based in a single chip containing two internal replicate probe-sets (2×) of eleven probes per unigene, covering completely the 350K spotted platform. Thereby, we generated a high-density oligonucleotide-based microarray platform for transcriptome studies in melon.

**Table 1:** Melon Expressed Sequence TAG (EST) dataset used for microarray construction

**Library**	**Subspecies/cultivar/accession**	**Tissue/physiological condition**	**High quality ESTs**	**Mean EST length (bp) ESTs ± SD**	**Unigenes**	**Redundancy (%)**	**Library specific EST**
15d	Ssp. melo cv. "Piel de Sapo" T111	Fruit 15 days after pollination	3582	608.1 ± 175.2	2939	18	1100
46d	Ssp. melo cv. "Piel de Sapo" T111	Fruit 46 days after pollination	3493	583.0 ± 161.1	2854	18	1063
A	Ssp. Agrestis accession pat81	Healthy roots	3666	700.0 ± 185.4	3189	13	1365
AI	Ssp. Agrestis accession pat81	Roots infected with M. Cannonballus	3255	756.3 ± 137.1	2616	20	1005
CI	Ssp. melo var. Cantaloupe accession C-35	Cotyledons infected with CMV	5664	651.4 ± 205.7	4679	17	2264
HS	Ssp. melo var. Cantaloupe accession C-35	Healthy leaves	3012	669.3 ± 171.1	2548	15	998
cm	Ssp. melo var. charentais	Leaves	11	597.5 ± 74.1	11	0	8
f	Ssp. melo cv. "Piel de Sapo" T111	Fruit immature	206	412.4 ± 174.4	190	8	70
mc_fi	Ssp. melo cv. "Piel de Sapo" T111	Fruit immature	106	610.8 ± 119.6	99	7	49
mc_p	Ssp. Agrestis accesion PI 161375	Seedlings	748	565.2 ± 135.8	623	17	268
PS	Ssp. Melo cv. "Piel de Sapo" Piñonet torpedo	Healthy roots	3377	679.9 ± 198.7	2945	13	1258
PSI	Ssp. Melo cv. "Piel de Sapo" Piñonet torpedo	Roots infected with M. Cannonballus	3555	749.3 ± 156.2	3105	13	1363
cornell_fr	Ssp. Melo var. "tam_Dew	Mature fruits	2783	529.7 ± 159.1	1922	31	733

To test the microarray platform, we designed three different experimental set ups. First, we compared transcriptional changes of (1) fruit development, using immature fruits (15 days after pollination, namely 15d) vs. mature fruits (46 days after pollination, 46d) in the non-climacteric Piel de Sapo T111 cultivar; (2) *C. melo *ssp agrestis Pat81 (resistant to the fungus responsible for melon root/vine decline, *Monosporascus cannonballus*, henceforth *M.c.*) after inoculation with *M.c. *(AGI) vs. non-infected roots (S) and, (3) cotyledons infected with *Cucumber Mosaic Virus *(CMV) inoculated in the Piel de Sapo tendral cultivar (S) vs. a mock inoculation (M).

### Microarray quality testing and normalization

To evaluate the microarray quality, three biological replicates were hybridized for each experiment. Technical replicates, as well as internal replicates, were also carried out in order to check the reliability of the hybridization and the precision of the microarray. After hybridization and array scanning, images of the physical hybridization were visually inspected for artefacts such as scratches, bubbles, and high regional or overall background. Detected pixels or other non-uniformities in a scanned image were determined as outliers and thus excluded from downstream analyses (data not shown).

**Figure 1:**
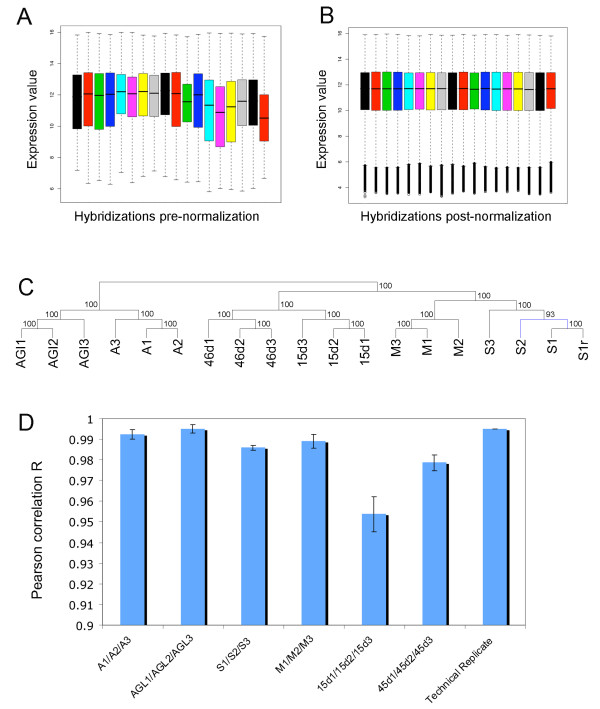
**Quality analyses between biological and technical hybridization replicates**. Box-plots of the samples before **(A) **and after **(B) **the normalization step. The baseline is set to a similar raw expression level, allowing the inter-chips comparison. **(C) **Hierarchical clustering (HC) of the samples performed using the whole expression data after normalization. Different conditions are separated and replicates cluster together. **(D) **Pearson correlation at gene-level for all the probes in the replicates of the microarray. All the replicates showed a correlation value greater than p > 0.95 thus showing a high level of similarity.

Replicates were further taken into a normalization step using the R statistical software [33] and the *oligo *package [34] applied for high-density oligonucleotide Nimblegen^® ^one-colour microarrays within the Bioconductor project of open source software [35]. The expression intensities were calculated from scanned images and normalized using the Robust Multichip Average (RMA) method. Box plots of both pre- (Figure 1A) and post-normalization (Figure 1B) confirmed that our data were successfully normalized. Data quality was assessed comparing the signal intensity data from each array to that obtained from the technical or biological replicates. Pearson correlation between replicates was calculated for every gene in all the arrays, resulting in a very high correlation level, with a coefficient >0.95 for every independent experiment in a pairwise comparison (Figure 1D). This high coefficient is indicative of the precision level in which the microarray is able to process transcriptomics data reliably.

A support tree clustering method with bootstrapping (ST) and a principal component analysis (PCA) using expression data were performed to statistically validate the tool. ST is an improvement of the classical hierarchical clustering method (HCL) [36] implementing statistical support for the nodes of the trees based on resampling the data, in our case by bootstrapping from 100 iterations. By performing this statistical analysis, which is used to evaluate the reliability of a tree, we assessed a high level of similarity between the replicates (Figure 1C).

**Figure 2:**
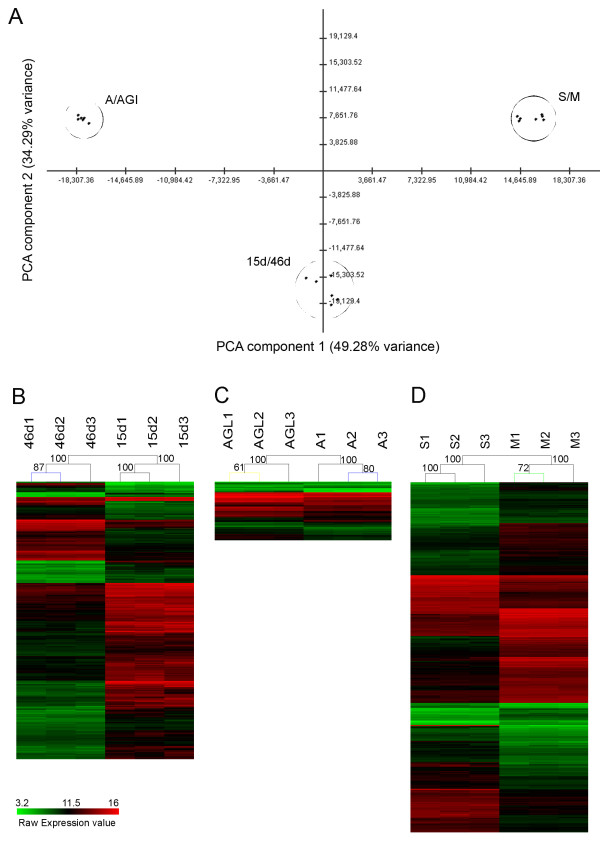
**Analyses of differential transcription for the three different data set used in the microarray**. **(A) **Principal Component Analysis (PCA) of the transcript profiles from all the melon samples in the microarray, showing a high separation for each experimental set up. The array data used for this analysis were normalized using the RMA algorithm implemented in the *oligo *R package for Nimblegen^® ^arrays. Pannels (B), (C) and (D) display support trees (ST) of the deregulated genes for all the conditions. **(B) **ST of the 937 deregulated genes with a q < 0.01 using the SAM in the 46d vs 15d melon fruit. **(C) **ST of the 198 deregulated genes with a q < 0.01 using the SAM in the *M. cannonbalus *infection. **(D) **ST of the 1182 deregulated genes with a q < 0.01 using the SAM in the CMV infection.

The application of PCA to expression data (where the experimental conditions are the variables, and the gene expression measurements are the observations) let us summarize gene set expression variation in the different conditions [37]. PCA analysis results together with the ST clustering results showed that the three different experimental populations showed differential expression patterns (Figure 2A). Overall, the statistical analyses showed significant separation of the three experiments, with the biological replicates clustering together (Figure 1C; 2A). These results were consistent with the high precision level observed by correlation analysis, supporting the high quality of the hybridizations. Together, these studies corroborate the ability of our tool for comparing different experimental conditions.

Taken together the statistical analyses, we conclude that the microarray platform was efficiently generated, hybridized and normalized.

### Detection of differentially regulated genes

After normalizing the microarray data, we identified the statistically deregulated genes by using the Significance Analysis of Microarrays (SAM) method [38] and its implementation in the MultiExperiment Viewer (MeV v4.2) software suite [39]. A total of 937, 198 and 1182 genes appeared significantly and differentially regulated during fruit ripening, *M.c. *and CMV infection experiments respectively. Surprisingly, we observed a high variation on differential gene expression inference between the two-pathogen infections (Figure 2C-D). A putative EST density bias due to higher unigene representation for CMV infection is an unlikely explanation, as a similar magnitude of differentially expressed genes was found both in our analysis and previous studies on CMV infection [40]. Besides, our microarray has a high percentage of pathogen-interaction EST probes. Rather, the low differentially gene expression found in the *M.c *experiment could be due to biological issues such as the semi-resistant phenotype of the pat81 line, as suggested in González-Ibeas et al. [1].

Different fold-change (FC) thresholds were determined according to the SAM analyses for each experiment: FC > 2.1 and FC < 0.53 for 46d vs 15d; FC > 1.3 and FC < 0.75 for AGI vs A; FC > 1.67 and FC < 0.56 for S vs M samples (see methods). These results further support the evidence that *M.c *infection on the particular cultivar tested induces less and more subtle changes compared to the other conditions.

**Figure 3:**
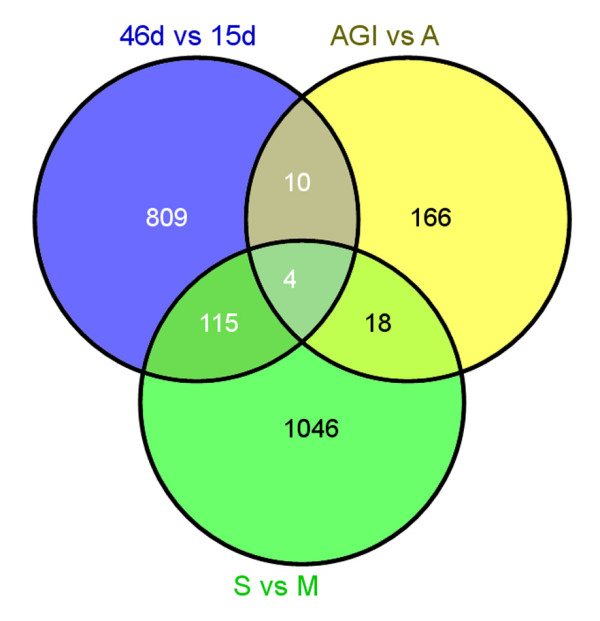
**Venn Diagram addresses the high experiment-specificity of the microarray**. Venn Diagram using all deregulated genes for all the experiments showed that little overlapping exists. Only four genes were shared between the three experiments, and overlapped genes in pairwise comparison did not exceed 10% of total deregulated genes.

Interestingly, a Venn-Diagram for all experiments showed that 83.8% of differentially regulated genes during fruit ripening were specific for that condition (809 genes) when comparing it with the other microarray hybridization experiments done in this study (Figure 3). The same was observed in the other two experiments, namely 86.7% in *M.c. *infection (166 genes) and 88.5% in CMV infection (1046 genes) (Figure 3). This high specificity indicates that the microarray platform is able to detect changes in expression profiles both during fruit development or pathogen interaction with high precision with no bias effect due to the probe representation design.

Next, to determine the trend in gene expression for each experiment and to visually display it in two dimensions, we generated a heat map taking into account only genes that showed significant changes in gene expression. The Support Tree (ST) hierarchical clustering method applied to the heat map separated all genes by experiment and condition (Figure 2B-D). This analysis showed a clear trend to down-regulation of gene expression during fruit ripening (Figure 2B) with 74.6% of down-regulated genes and 25.4% of upregulated genes. In the case of *M.c *infection, we observed that 31.3% of the genes showed down-regulation and 69.19% were upregulated (Figure 2C). After CMV infection, 58.46% of the genes were upregulated and 41.62% were downregulated (Figure 2C). A list showing all of the genes differentially regulated for each experiment (FDR = 0) is provided in Additional files 1, 2 and 3

### Quantitative real-time PCR validation

To validate the microarray data and evaluate the accuracy of the platform, we performed quantitative real-time PCR (qRT-PCR) on 12 genes. Four genes for every microarray experiment were selected based on previous bibliographic reports and deregulation levels. Then their expression profiles were obtained (Table 2). The oligonucleotide sequences used are summarized in Table 3. The correlation between the microarray results, and those obtained by qRT-PCR was assessed by calculating the Pearson's product moment correlation coefficient [41,42] at replicate level (Table 2). This test statistically assigns a correlation coefficient to the difference in the fold-change from both microarray and qRT-PCR techniques. A global correlation coefficient of 0.868 calculated by the average of every gene was observed. These results indicate that our microarrays are able to detect both low and high fold-changes with high accuracy in different experimental conditions (p-value < 0.01) (Table 2).

**Table 2:** Microarray and qRT-PCR results of the 12 selected genes with their replicate-level Pearson correlation

**Gene Identifier**	**Microarray**	**qRT-PCR**	**Correlation**	**p-value**	**Samples**
cCL1715Contig1	10,807	13,279	0.999	1.23E-06	S vs M
cCL3206Contig1	18,273	25,442	0.969	1.35E-03	S vs M
c15d_08-H10-M13R	47,246	138.50	0.988	2.00E-04	S vs M
cPSI_02-F08-M13R	15,915	37,523	0.998	2.84E-06	S vs M
cCL2301Contig1	2,316	2,140	0.889	1.77E-02	AGI vs A
cPSI_33-C12-M13R	2,564	2,370	0.888	1.79E-02	AGI vs A
cCL3647Contig1	-3,330	-2,941	0.696	1.24E-01	AGI vs A
cCL1700Contig1	-1,581	-1,563	0.781	6.66E-02	AGI vs A
cCL3137Contig1	-57.16	-105.80	0.823	4.43E-02	46d vs 15d
cCl5879Contig1	373.92	947.39	0.843	4.58E-04	46d vs 15d
cCL451Contig1	-32.75	-12,367	0.916	1.04E-02	46d vs 15d
c46d_34-C03-M13R	12.02	23,314	0.633	3.07E-03	46d vs 15d

**Table 3:** Genes and primers used for quantitative RT-PCR

**Gene**	**Forward primer (5"-3")**	**Reverse primer(5-3')**
cCL5879Contig1	AACTTTTTGTGAGTGTGTAATCGTTTTATA	CCGAACATAATGTTACGAATCGATAT
cCL451Contig1	ATAGTAATAAGGAATATTAGAGGGCTTGTGT	ACCCACTTAAAAAGGGCAAACA
46d_34-C03-M13	CGAAGGGATGAAATTTGTTTGTAAGAACTAAT	CCATTTTTGGTTCATATATAGAAA
cCL3137Contig1	ATGATATTATTATTCGAAATTGGGAAGTG	AGCAGTCTTGTCTTTTGCTTCTCA
cCL1715Contig1	TAGTTGGTG TGGACCGTGTAGAA	CAGTGTCGGTGTTGAGCACAA
cCL3206Contig1	GCCTTTCGCCCTTCACTTAA	GGAGAAGAAGGCAGCTTATGCTT
c15d_08-HlG-M13R_c	TTATCGTCTTTATGCCCCGAGT	GGTTTCGTTGTCCACTTGATTTT
cPSI_02-F08-M13R_c	TCTTCGAATGTGGTGGGTTCA	CAAAGGCGGTGAATCGAGAA
cCL1700Contig1	TAATCGGTAAGGACGGTTCTG	TAATCGGTAACGACGGTTCTG
cCL230lContig1	TCGCTCGACTTGATGAAAGAT	AGGTG AAATTCCCTCCTTCAA
cCL3647Contig1	GAGTGGATGGATGAGGAAATG	AAGTTCCAGGCTTAACCCAAA
cPSI 33-C12-M13R	ACTCGATCAACTTCGAGCAAA	TCCCACTGAAGAATACGCATC

### Functional analysis of microarray results

In order to shed light into the processes involved in the studied conditions, we analyzed for enrichment of Gene Ontology (GO) terms among genes differentially expressed. GO annotations were provided by González-Ibeas et al. [1] (see Methods). This analysis highlights biological processes that have statistically significant higher number of genes differentially expressed in the studied conditions. Results for GO enrichment analysis and validation by qRT-PCR of selected genes for each independent experiment are explained below.

#### Fruit ripening involves deregulation in ethylene signalling, sugar metabolism and cell wall-loosening enzymes

**Figure 4:**
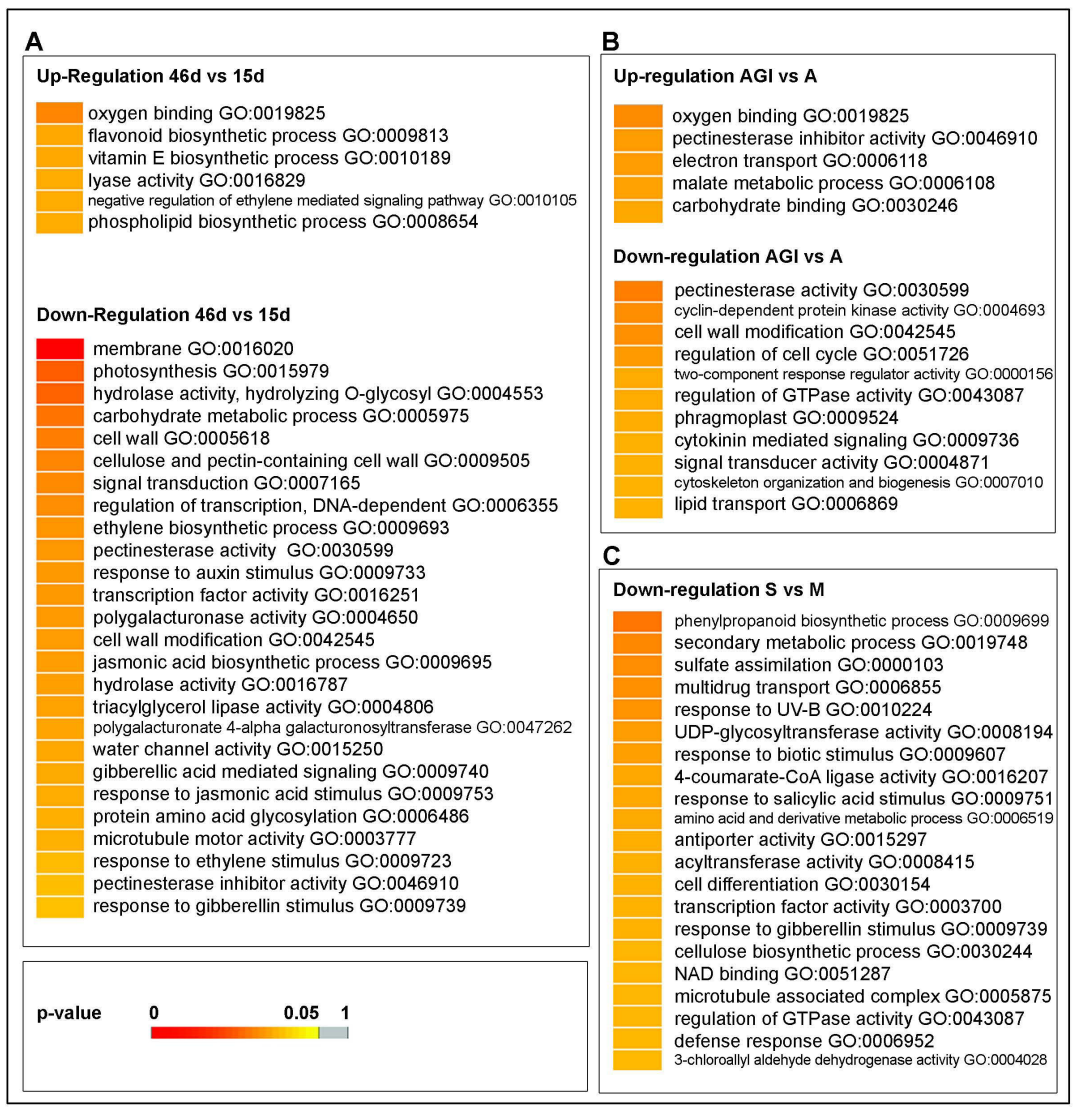
**Gene Ontology functional enrichment**. **(A) **Fruit ripening experiment was analyzed for functional enrichment using Gene Ontology terms and Z-score statistics calculation (see Methods). Resulting p-values after FDR multiple testing adjustments are visualized in a colour code scale; colours toward red signify enrichment for a particular GO term. Gray means no statistically significant enrichment. **(B) **Enrichment analysis, using Z-score statistics, of the *M. cannonbalus *infection in the Agrestis accession roots. **(C) **Enrichment analysis of the CMV infection in photosynthetic cotyledons in *C. melo *var. tendral.

Several GO terms related to cell wall metabolism were enriched between samples 15d and 46d (Figure 4A). These included "cell wall", "cellulose and pectin-containing metabolic process", and both polygalacturonase and pectinesterase activities (Figure 4A). These are indicative of the important cell-wall modifications associated to the fruit ripening process.

We further corroborated these changes in gene expression during fruit ripening at gene-level and validated the microarray results by qRT-PCR for four selected individual genes that appeared enriched in these categories. Three genes putatively involved in carbohydrate metabolism, cell-wall softening and ethylene production with differential expression between immature (15d) and mature fruit (46d) of the non-climacteric melon *Piel de Sapo *T111 were selected for qRT-PCR validation. cCL3137Contig1, is similar to a proline-rich cell wall protein (AT3G22120, 56% similarity, E<1E-15) which was dramatically shut-down in mature fruits (FC = -105.8) (Table 2). In fact, in fleshy-fruit species the flesh softening process implies disassembly and reorganization of the cell walls during the fruit ripening process [43].

As determined by the functional enrichment, "carbohydrate metabolism" related genes were differentially expressed during ripening (Figure 4A). Thus, a number of cell wall invertases appeared deregulated (Figure 4A), while others appear upregulated. We chose c46d_34-C03-M13R, which is highly similar to an invertase (UniRef90_Q9ZR55, 64% similarity, E<3E-48) for qRT-PCR validation (FC = 23.314) (Table 2), again confirming our overall observations.

Interestingly, although Piel de Sapo T111 is a non-climacteric variety, we found enrichment for ethylene-related GO terms in the fruit ripening process, showing an over-representation of negative regulation of ethylene-mediated signalling in the upregulated genes, whereas "ethylene biosynthesis" and "response to ethylene categories" were enriched in down-regulation (Figure 4A). These results provide a molecular insight for the non-climacteric fruit ripening nature of the melon cultivar PS used in our study.

Moreover, the cCL451Contig1 was selected for validation due to its homology to the CmACO1 (1-aminocyclopropane-1-carboxylate oxidase I) [44], involved in ethylene synthesis (UniRef90_Q04644, 88% similarity, E<1E-158). It was shown that CmACO1 antisense expressing plants showed no ethylene production and extended shelf-life in the climacteric Cantaloupe cultivar, which is of high economic importance [45]. Interestingly, we found that CmACO1 was under-expressed in mature fruits 46d (FC = -12.36) (Table 2), supporting a differential role of this gene controlling ethylene production in climacteric and non-climacteric fruits.

Finally, to address the detection range of the microarray we chose the most deregulated gene during fruit ripening in our experiment for qRT-PCR validation. The cCL5879Contig1, similar to a gene encoding for a serine-threonine kinase like protein (AT1G01140, 52% similarity, E<1E-12) that showed a FC = 947.39 (Table 2). The homologue of this gene was described as an interactor of calcineurin B-like calcium sensor response proteins (AtCBLs) in Arabidopsis [46]. CBLs are implicated in signalling pathways in response to stress, hormones and environmental cues [46], together supporting an important hormonal regulation of fruit maturation in melon.

Overall, our results indicate a high reproducibility level of the dynamic range between the microarray data and the expression profiles by qRT-PCR.

#### Responses to fungi involve deregulation of signal transduction pathways and cell wall and cytoskeleton rearrangements

GO enrichment analyses showed the preferential over-expression of genes related to terms such as "pectinestarase inhibitor activity" and "oxygen binding", related to cytochrome P450 (Figure 4B). It was reported that over-expression of pectin methylesterase inhibitors in *Arabidopsis *restricts fungal infection [47]. It is also well known that pathogen infection usually causes oxidative bursts, triggering the accumulation of reactive oxygen species (ROS) as an early response [48,49]. Accordingly, GO statistical analyses showed enriched over-expression of genes involved in electron transport (Figure 4B), as expected from known responses [48].

Moreover, "cell wall modifications", "cytoskeleton organization and biogenesis", and cell cycle related GO terms such as "cyclin-dependent protein kinase activity", were statistically enriched in downregulated genes, indicating specific changes in these transcriptional responses (Figure 4B).

The expression of four differentially regulated genes involved in "cell wall modifications", "actin cytoskeleton modification" and "oxidative stress" was validated by RT-PCR. The cCL1700Contig1, a putative profilin protein homologue to Arabidopsis AT2G19760 (PRO1, 74% similarity, E<1E-53) was verified by qRT-PCR (FC = -1.536) (Table 2). Profilin is the main monomer actin binding protein in plant cells [50]. Recent studies suggested that profilin is a multifunctional protein with antagonistic effects on actin polymerization. Previous studies related changes in profilin expression in response to *Phytophtora infestans *[51] in parsley, and to *Tilletia tritici *or *Fusarium gramineum *in wheat [52,53]. In agreement, the down-regulation of cCL1700Contig1 in *M.c. *infected roots support the role for profilins in cytoskeleton reorganization during the defence response in melon. These results were consistent with the determined key role of cell wall and cytoskeleton rearrangement in fungal defence [54].

The unigene cCL2301Contig1 is a putative melon orthologue of PcCMPG1 from parsley (*Petroselinum crispum*) (76% similarity, E<1E-129), Arabidopsis AT5G37490 (65% similarity, E<8E-99), [55] tobacco NtCMPG1 (71% similarity, E<1E-114) and tomato SlCMPG1 (70% similarity, E<1E-106) [56]. These have been reported as fast-response genes to elicitor treatment and hence are considered mediators of the immediate early response in *Petroselinum crispum *[57]. Our qRT-PCR results validated the observed up-regulation after inoculation in melon agrestis Pat81 accession (FC = 2.140) (Table 2), indicating that this gene might be involved in the response to fungal attack in this resistant melon line [58]. This result was consistent with a previously reported effect of increased resistance in CMPG1 over-expressing tobacco, while silencing of this gene led to a decreased resistance [56].

cCL3647Contig1 is a putative orthologue to Arabidopsis AT2G21610 (81% similarity, E<1E-121), a member of the pectinesterase protein family [59]. These enzymes carry demethyl-esterification of pectins, a main component of the primary cell wall. Changes in the degree and pattern of methyl esterification of pectins are associated with cell wall modifications and can influence root development and responses to biotic and abiotic stresses [60]. We found a decreased expression of cCL3647Contig1 (FC = -2.941) (Table 2) after *M*.*c *infection suggesting that pectin biosynthesis might be regulated during defence response in melon.

#### CMV infection induces structural and cell-cycle deregulation

It has been previously described that plant viruses transiently suppress host gene expression [61]. In accordance, our analysis showed that 58.46% of deregulated genes were under-expressed in response to CMV infection. Indeed, our GO analysis did not show any category statistically upregulated (Figure 4C), though less stringent analyses pointed to several GO categories potentially upregulated (data not shown). Perhaps strikingly, down-regulated categories include defence-related terms, such as "phenylpropanoid biosynthetic processes" and "defence response" (Figure 4C). Despite this apparent general host gene suppression effect, specific transcripts related to defence responses after pathogen attack increased significantly in their accumulation. These include, for example, WRKY transcription factors and beta-glucanases. To validate these data, we performed qRT-PCR with four CMV-infection related genes. The unigene c15d_08-H10-M13R_c, highly similar to PR-1-like protein in Arabidopsis (AT4G25780, 83% similarity, E<3E-64), showed a strong induction (FC = 138.50) (Table 2). The unigene cPSI_02-F08-M13R_c, which is highly similar to the elicitor-inducible gene EIG-I24 of tobacco (UniRef90_Q9FXS7, 73% similarity, E<1E-72) and is upregulated after treatment with hyphal wall components [62], was induced in CMV infected cotyledons (FC = 37.523) (Table 2). Interestingly, c15d_08-H10-M13R_c and cPSI_02-F08-M13R_c genes were not upregulated at significant level after *M.c. *infection in melon pat_81 (an accession resistant to this fungus).

Microarray analyses revealed significant changes in gene expression of at least four circadian clock genes. In particular, the cCL3206Contig1, highly similar to a pseudo-response regulator (APRR5) whose mutation in Arabidopsis (AT5G24470, 78% similarity, E<5E-21) exhibited circadian-clock associated phenotypes [63], was confirmed by qRT-PCR to decrease its accumulation in virus-infected plants. Inhibition of photosynthesis, chloroplast damage, and reduction of chloroplast proteins levels are usual alterations in many virus infections, and oxidative stress has been described to be involved in several plant-virus interactions, including CMV infections [66]. The unigen cCL1715Contig1 is highly similar to AT2G15570 gene (67% similarity, E<7E-43), a chloroplast type 3 thioredoxin with thiol-disulfide exchange activity and involved in cell redox homeostasis [64]. Downregulation of this gene was also validated by qRT-PCR (FC = 13.279) (Table 2).

## Conclusion

We report the construction and validation of a high-density oligonucleotide-based microarray tool for functional genomics studies in melon. Our results reveal that this new tool is amenable for high-throughput gene expression analyses in different melon cultivars. Furthermore, the use of the array has proven to be valid for genomic studies on different plant tissues, developmental stages as well as in a range of biotic-stress responses. As a proof of principle, we report changes in gene expression generated by this microarray in three independent experimental set ups. The statistical analyses of our microarray data and the functional analyses using the Gene Ontology annotations served to characterize the gene expression changes in the validated experiments. These results not only validate our chip, but also provide an important molecular view in yet undocumented ripening and defence responses in melon.

Overall the novel melon microarray platform offers the possibility to carry functional genomics studies of fruit-quality traits in melon, and will be very valuable for those researchers interested in *Cucumis melo *transcriptomics.

## Methods

### Plant material

The material used for the transcriptomic profile analyses came from three different *C. melo *accessions: the line T-111, which corresponds to a Piel de Sapo breeding line, the Tendral variety (Semillas Fitó, Barcelona, Spain), and the pat81 accession of *C. melo *L. ssp. *agrestis *(Naud.) Pangalo maintained at the germplasm bank of COMAV (COMAV-UPV, Valencia, Spain). Seeds of T-111 were germinated at 30°C for two days and plants were grown in a greenhouse in peat bags, drip irrigated, with 0.25-m spacing between plants. Fruits were collected 15 and 46 days after pollination and mesocarp tissues were used for RNA extractions. Roots from pat81 plants were mock treated or inoculated with 50 colony-forming units (CFU) of *M.c*. per gram of sterile soil and harvested 14 days after inoculation. Cotyledons from var. Tendral, mock treated or mechanically inoculated with CMV, were harvested 3 days post inoculation. Plant growth and infections were done as described in González-Ibeas et al. [1].

### RNA Isolation and cDNA preparation

Total RNA from each of the tissues was extracted using Plant RNeasy Mini Kit (Qiagen, Hilden, Germany) following the manufacturer protocols. The resulting RNA is enriched in molecules longer than 200 nucleotides. DNA contaminations were removed using the DNA-*free*™ Kit (Ambion, Austin, TX) according to the manufacturer instructions. The RNA quantity and purity were determined by Nanodrop ND-1000 spectrophotometer (Nano Drop Technologies, Wilmington, Delaware). Integrity and quality of the RNA were checked by agarose electrophoresis. Only those high-quality RNA samples were reverse transcribed into cDNA using the SuperScript™ Double-Stranded cDNA Synthesis Kit (Invitrogen, Carlsbad, CA). cDNA integrity was assessed with an Agilent Bioanalyzer 2100 and RNA 6000 NanoLabChip Kit (Agilent Technologies, Palo Alto, CA).

### Design and production of the melon Nimblegen^® ^custom array

A dataset of 17,510 quality-filtered unigenes from different normalized cDNA libraries were submitted for probe design and production of the high-density microarray (Table 1). The Nimblegen Maskless Array Synthesis (MAS) technology was used to fabricate the microarray, combining photo-deposition chemistry with digital light projection. Our design is based on a single chip containing two internal replicate probe sets (2×) of 11 probes per unigene and controls such as random GC oligonucleotides, covering the whole 385K 1-plex platform spots. Thus, every unigene is represented by an average of eleven 60-mer probes, synthesized *in situ *by photolithography on glass slides using a random positional pattern. The melon microarrays are now commercially available at Nimblegen^®^.

### Microarray probe preparation

Long oligonucleotide probes generated for this novel *C. melo *microarray (60 mers) provide superior signal-to-noise ratio, increased sensitivity, specificity, and discrimination. Probes were designed taking into account characteristics such as non-repetitive elements, frequency, uniqueness and melting temperature (Tm). Highly repetitive elements of the transcript set were excluded using a method developed by Morgulis (2006) that identifies these regions and exclude them from probe selection [65]. Each oligonucleotide is then compared to the transcriptome and the similarity is reduced to a Boolean value based on the weighted mismatch score. This score is compared to a set threshold (usually 10) and if the score is higher, it is considered as unique. The Tm and the self-complementarities of the 60 mer oligos are also ranked and those with higher scores were selected as probes.

### Microarray hybridization and quality control

The synthesized cDNA (1 μg) from each sample was transcribed to cRNA and labelled with cyanine 3 (Cy3)-labelled nucleotides following Nimblegen^® ^specifications. Hybridization signal intensity (HSI) was calculated using a GenePix 4000B Scanner and associated software. Quality controls such as visualization of hybridization images for finding artefacts or high HSI and background zones were visually performed. RNA digestion plots to know the amount of RNA degradation that occurred during its preparation and the quality of the second strand synthesis were performed using Perl scripts.

### Normalization and statistical analysis

The intensity values obtained from the array scanning were normalized using the *oligo *package for the R statistics software. The workflow used to normalize our data was followed as explained by the package provided for Nimblegen^® ^expression microarrays. An automatic pipeline using Perl and R scripts was generated to map every probe to its gene and normalize the data using the Robust Multichip Average (RMA) algorithm [66]. It consists of three steps: a background adjustment, quantile normalization and finally log transformed PM values summarization [67]. It has been reported that RMA gives the most reproducible results and shows the highest correlation coefficients with qRT-PCR data on genes identified as differentially expressed [68].

After normalization, a hierarchical clustering support tree method was performed using TMeV 4.0 [39] software from TIGR. The Euclidean distance was used as a measure of similarity or distance between samples. As a rule for the linkage of clusters, the average linkage method was used. In this method, distance calculation is based on the average distance between objects from the first cluster and objects from the second cluster. The averaging is performed over all pairs, determining the actual distance between two clusters.

To statistically infer the deregulated genes in the microarray, the Significance Analysis of Microarrays (SAM) algorithm was run using the TMeV 4.0 software. Multiple testing adjustments for False Discovery Rate (FDR) method, based in Benjamini and Hochberg's method [69] were performed, forcing it to be FDR≤0, therefore allowing for a highly stringent analysis with no false positive identification of differentially regulated genes. This methodological approach resulted in a variable SAM delta value (δ) - which defines the threshold of false positive in the validated dataset-, depending on the experiment. The Venn Diagram was generated using the online Venny tool [70] The correlation between the microarray and qRT-PCR data was assessed through Pearson's moment correlation analysis. R software package was used to systematically test all the expression changes of the qRT-PCR validated genes. Correlation analyses were also performed in order to check for similarities between the biological and technical replicates.

### Gene Ontology functional enrichment analysis

Functional annotation of genes based on Gene Ontology was provided by González-Ibeas et al. [1]. We used an inclusive analysis for differentially expressed genes, taking into account the GO hierarchy, in which genes annotated with terms that are descendent of the correspondent term in a specific level, obtain the annotation from the parent.

To infer the statistical enrichment we used a similar method and visualization approach as in [71]. In particular, enrichment was assessed using binomial distribution and p-value was calculated as  where: x = number of differentially expressed genes in the category, n = total number of genes in the category and p = frequency of upregulated or downregulated genes. Resulting p-values were adjusted for multiple testing using the Benjamin and Hochberg's method of False Discovery Rate (FDR) [69]. Statistical results were visualized in a scale-based colour gradient depending on the p-value. Red indicates enrichment and gray indicates no statistical significance after FDR adjustment.

### Quantitative real-time PCR

To validate the expression changes found in the microarray experiments, transcript levels of the 12 selected genes transcripts were quantified by the ABI Prism 7700 (Applied Biosystems, Foster City, CA, U.S.A). The oligonucleotides chosen to amplify the selected genes were designed using the Primer Express Software (Applied Biosystems). Detection of the PCR products was performed as explained by the manufacturer using the Power SYBR green dye (Applied Biosystems) and ROX as passive reference. To calculate the relative change in expression levels, we used the Data Analysis for Real-Time PCR (DART) software with three technical replicates for statistical analysis [72]. Melting curves analyses at the end of the process and No Template Controls (NTC) were carried out to ensure product-specific amplification and no primer-dimer quantification. A control reaction without reverse transcriptase was performed to evaluate genomic DNA contamination. Endogenous controls were performed using relative quantitative accumulation of Cyclophilin (cCL1375) levels in every condition.

### Availability of the microarray data

Microarray data are publically available at Arrayexpress . The Experiment name for to the reported hybridizations is "melogen_melo_v1", ArrayExpress accession E-MEXP-2334.

## Appendix 1

Current address of S.M-G: Fundación Instituto Leloirand IIBBA-CONICET, Av. Patricias Argentinas 435, C1405BWE, Buenos Aires, Argentina.

## Authors' contributions

AMC, JV, SMG, WD, DGI and AICD prepared RNAs, cDNAs for microarray, and JV. CR, MS, DGI carried out the Real-Time-qPCR experiments. AMC, JB and JC carried out the bioinformatics analyses. PP is the co-ordinator of The MELOGEN initiative and participated in the conception of the study together with JGM, MAA, BP, FN, NLB and ACD ACD is the principal investigator of this study and responsible for the delivery of a melon Microarray tool. AICD and AMC processed all the data and wrote the manuscript. All authors read and approved the final manuscript.

## Supplementary Material

Additional file 1 **Table S1 - List of deregulated genes during fruit ripening (FDR = 0)**. The data provide correspond to the list of melon genes whose expression is modified from 15d to 40d ripening. Click here for file

Additional file 2 **Table S2 - List of deregulated genes after Monosporascus cannonballus infection (FDR = 0)**. The data provide correspond to the list of melon genes whose expression is modified after fungi infection. Click here for file

Additional file 3 **Table S3 - List of deregulated genes after CMV infection (FDR = 0)**. The data provide correspond to the list of melon genes whose expression is modified after virus infection. Click here for file
